# Development and Validation of a Model to Predict Secondary Arrhythmia in Patients With Epilepsy

**DOI:** 10.1111/cns.70670

**Published:** 2025-11-26

**Authors:** Yulong Li, Zhen Sun, Shen Su, Jun Zhao, Yanping Sun

**Affiliations:** ^1^ Department of Neurology The Affiliated Hospital of Qingdao University Qingdao China; ^2^ Department of Gastroenterology The Affiliated Hospital of Qingdao University Qingdao China

**Keywords:** arrhythmias, epilepsy, prediction model

## Abstract

**Objective:**

Compared with healthy individuals, epilepsy patients are more prone to arrhythmias, which may contribute to poor prognosis. To enable early identification of this risk, we developed a clinical prognostic prediction model to assess the risk of arrhythmia comorbidity in epilepsy patients, thereby facilitating timely clinical intervention to improve patient outcomes.

**Methods:**

We retrospectively collected clinical data from epilepsy patients treated at the Affiliated Hospital of Qingdao University between January 2022 and February 2025, including gender, age, medical history, antiseizure medications, electrocardiograms and electroencephalograms. A total of 495 eligible patients were enrolled and randomly divided into development and validation datasets at a 7:3 ratio. Variable selection was performed using LASSO regression with a penalty term, and the selected variables were incorporated into the construction of a logistic regression model. The area under the receiver operating characteristic curve (AUC) and its 95% confidence interval were used to preliminarily evaluate the model's discriminative ability, while cross‐validation and bootstrapping were employed to assess its generalizability. Calibration curves and the Brier score were utilized to evaluate the model's calibration, and decision curve analysis was plotted to analyze the net clinical benefit.

**Result:**

The C‐indices for the development and validation datasets were 0.737 (95% CI 0.675–0.799) and 0.790 (95% CI: 0.707–0.884), respectively, with an overall C‐index of 0.752 (95% CI: 0.701–0.804). The corresponding sensitivity and specificity were 74.6% and 68.1%, respectively. Finally, a nomogram was constructed for the visual presentation of the predictive model.

**Conclusion:**

Our predictive model can accurately assess the risk of arrhythmia comorbidity in epilepsy patients, assisting clinicians in early intervention to improve prognosis.

## Graphical abstract

This graphical abstract visually synthesizes how multimodal clinical data feeds into a validated predictive algorithm for proactive arrhythmia monitoring in epilepsy care.

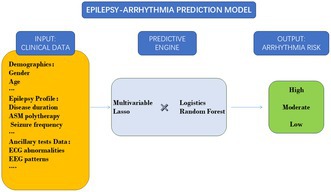

## 1 Introduction

Epilepsy is one of the most prevalent neurological disorders. A growing body of epidemiological and correlational evidence indicates a strong association between epilepsy and arrhythmias [1, 2, 3]. Patients with epilepsy (PWE) have a higher risk of arrhythmias than healthy people. Some malignant arrhythmias can even lead to sudden unexpected death in epilepsy (SUDEP) [4], which is the leading cause of epilepsy‐related mortality in both children and adults [5, 6]. Epilepsy and arrhythmia can mutually interact in various ways [7, 8]. Focusing on the underlying arrhythmias of PWE and intervening early may save their lives because some easily overlooked arrhythmias may be a distress signal from the body.

The correlation between epilepsy and arrhythmia can be categorized into several distinct mechanisms. First, epileptic seizures directly affect cardiac electrophysiology [1]. A number of studies have demonstrated that stimulating parts of the brain has an effect on heart rhythm through autonomic transmission; for example, stimulation of the left insular cortex causes the heart rate to speed up, whereas stimulation of the right side results in a slower heart rate [1]. Research on patients with temporal lobe epilepsy has indicated that autonomic dysfunction is a primary cause of increased cardiac stiffness [9]. Furthermore, seizure‐induced hypoxaemia and sustained catecholamine elevation have been implicated in causing cardiac electrophysiological and mechanical dysfunction. The heightened risk of myocardial infarction (MI), arrhythmias, and sudden death observed in PWE supports the hypothesis that epilepsy can precipitate secondary cardiac dysfunction and increase mortality risk [10]. Second, certain antiseizure medicines (ASMs) exert proarrhythmic effects by modulating ion channels crucial for cardiac electrophysiology, with sodium channel blockers such as carbamazepine and valproic acid being most frequently reported [11, 12]. Conversely, some ASMs can also lower the seizure threshold [11]. Third, studies examining co‐expressed genes in epilepsy and arrhythmias have pointed out that mutations in some genes encoding both ion channels and non‐ion channels can predispose individuals to both epilepsy and arrhythmias, so‐called cardioembolic channelopathies [12, 13]. Finally, arrhythmias stemming from comorbidities in PWE may also elevate the likelihood of SUDEP, even when the arrhythmia is not directly caused by epilepsy.

SUDEP represents the most severe outcome for PWE. According to recent retrospective analyses, the incidence of SUDEP ranges from approximately 0.78‰ to 1.2‰, and appears to correlate positively with age [14]. Long‐term recurrent seizures are a primary risk factor, inducing physiological autonomic neuropathy. A substantial body of literature indicates that cardiac arrhythmias also contribute significantly to the pathogenesis of SUDEP [5]. Crucially, interictal arrhythmias are frequently overlooked, yet they may constitute a key pathophysiological substrate for adverse outcomes. Although there are currently no definitive methods for preventing SUDEP [15], regular cardiac monitoring in PWE is considered a promising risk mitigation strategy. While malignant tachyarrhythmias are rarely documented during seizures, various arrhythmias are detected in over 70% of PWE [16]. It can be speculated that arrhythmia may not be the sole direct cause of death of SUDEP; it likely acts as a critical precipitating or contributing factor of SUDEP [4]. Therefore, the identification of arrhythmic substrates during the interictal period could potentially reduce the incidence of SUDEP. The present study was conducted to develop predictive insights into arrhythmia risk in PWE, with the ultimate aim of facilitating timely interventions to improve patient prognosis.

## 2 Materials and Methods

### 2.1 Study Participants

This study retrospectively collected the medical records of patients diagnosed with epilepsy in the Affiliated Hospital of Qingdao University from January 2022 to February 2025. Inclusion criteria included: (1) Patients with a confirmed diagnosis of epilepsy; (2) a complete history of present illness, past medical history, and history of medication status of ASMs. Patients were excluded as follows: (1) suffered from cardiac arrhythmia before being diagnosed with epilepsy; (2) failure to take ASMs regularly. This includes not following medical advice regarding ASMs and frequently forgetting to take the medication (forgetting to take the medication more than twice a week); (3) a lack of complete or research‐suitable electrocardiogram (ECG) and electroencephalogram (EEG) data.

Outcome definition is arrhythmia present. Secondary Arrhythmia refers to cardiac arrhythmias that are diagnosed after the diagnosis of epilepsy, with no presence of arrhythmia prior to the epilepsy diagnosis.

### 2.2 Data Collection

Patients who developed arrhythmia were classified as the case group. The time of arrhythmia onset was designated as the index date. For each case, corresponding clinical data were collected from medical records preceding this index date, including gender, age, family history, history of stroke, traumatic brain injury, craniotomy of the brain and febrile seizures, ASM administration (type and dose), electrocardiograms (ECGs) and electroencephalograms (EEGs) results.

#### 2.2.1 Interpretation of Results: Analysis and Interpretation of ECG Data

A 12‐lead conventional ECG was recorded in 10 mm/mV and 25 mm/s. Lead V3R was not taken.

The acquisition of electrocardiogram (ECG) data is context‐dependent. ECG examinations are conducted in three settings: the emergency department, the ECG room, and the inpatient ward. ECG data obtained from the ECG room and the inpatient ward are considered valid ECGs, meaning they are suitable for research and analysis. ECGs acquired in the emergency department require further evaluation, as detailed below: If a patient has both an emergency ECG and an ECG from the ECG room/inpatient ward, the latter takes precedence. If a patient has only an emergency ECG, a valid ECG is defined as at least two emergency ECGs with consistent results. Otherwise, the patient is excluded from the study based on the exclusion criteria. The inclusion and exclusion criteria ensure that all study subjects have at least one valid ECG, acquired after the patient's first epileptic seizure. ECG results were interpreted according to the following criteria: (1) If all valid ECG results are normal, the outcome is classified as normal, and data are collected based on the time point of the most recent valid ECG; (2) If all valid ECG results show the same abnormality, the outcome is classified as abnormal, and data are collected based on the time point of the most recent valid ECG; (3) If there are two or more valid ECGs, with the first being normal and subsequent ones abnormal, the result is classified as abnormal. Data collection is based on the time point of the first abnormal ECG; (4) If there are two or more valid ECGs, with the first being abnormal and subsequent ones normal, the result is still classified as abnormal. Data collection is based on the time point of the first abnormal ECG; (5) For cases with multiple inconsistent valid ECG results, determinations were made on a case‐by‐case basis. Such cases were either excluded or explained in the results section. Respiratory sinus arrhythmia may be more common in adolescent and pediatric patients, and we adjudicated it as a normal ECG [17].

At the same time, we also extracted the PR interval, the QRS duration, the QT interval, the corrected QT interval and the frontal QRS‐T angle from the ECG [18].

The corrected QT interval was calculated using Bazett's formula: [QTc = QT/√(R‐R interval)]. The frontal QRST angle is calculated as the difference between the QRS axis and the T‐wave axis in the frontal plane. If the resulting angle exceeds 180°, it is adjusted by subtracting 360°. To avoid bias introduced by subjective measurements, automatically generated reports from the ECG machine were used to obtain the QRS and T‐wave axes. The use of objective machine‐derived data enhances the accuracy and reliability of the calculated frontal QRST angle [19].

#### 2.2.2 Antiseizure Medications

We have collected information on the use of ASMs by all of the patients. Each drug was included individually in the screening analyses of the factors. To avoid multicollinearity, individual drugs were included as separate factors in the analysis. Furthermore, classify whether the drug is taken, monotherapy or polytherapy. The inclusion doses for each drug in the study are as follows: Oxcarbazepine 600–2400 mg/day, Sodium Valproate 500–3000 mg/day, Lamotrigine 100–400 mg/day, Levetiracetam 1000–3000 mg/day, Carbamazepine 100–1600 mg/day, Lacosamide 100–400 mg/day, Perampanel 2–8 mg/day.

#### 2.2.3 Electroencephalograms

By reviewing the raw EEG data or EEG reports, our study categorized the EEG recordings into three groups: “normal EEG,” “abnormal background without epileptiform discharges,” or “epileptiform discharges.” All patients had interictal EEGs at the time of their visits, with monitoring durations ranging from 2 h to 24 h [20].

### 2.3 Statistical Analysis

For ensuring model stability, an exclusion criterion was applied to omit any independent factor that had a positive count ≤ 5.

Sample size calculation: This study included a total of 21 independent variables. According to relevant studies [21], the sample size for a risk prediction model should be at least 5–10 times the number of independent variables. In a preliminary sample of 50 cases, the incidence rate of arrhythmia was 28%. The required sample size for this study was determined to be at least 375. Furthermore, with 122 events observed in this study, the final number of variables in the model should be limited to fewer than 10 to ensure the stability of the prediction coefficients.

Categorical variables were expressed as percentages and continuous data were expressed as mean ± standard deviation (SD) or median and interquartile range (IQR).

To identify significant differences between groups, a preliminary univariate analysis was performed. Numerical data were compared using Chi‐squared or Fisher's exact test where appropriate, and continuous data were compared using the Mann–Whitney *U* test. A two‐sided *p* < 0.05 was considered significant.

LASSO regression was used respectively to screen the original variables. After performing LASSO regression on the variables, cross‐validation is performed. The lambda.1SE is selected and information about the selected variable is derived.

Subsequently, the overall data was divided into a development dataset and a validation dataset in the ratio of 7:3. Logistic regression is used as a modeling method. A logistic regression model was developed using the development dataset. The model subsequently underwent k‐fold cross‐validation and bootstrap optimism correction to evaluate its stability and generalizability. The Hosmer‐Lemeshow goodness‐of‐fit test to assess calibration with a *p* value of > 0.05 indicates good calibration [20].

Subsequently, the model performance was validated on the validation dataset, with the Area Under the Curve (AUC) and Precision‐Recall AUC (PR‐AUC) reported. Model calibration was assessed by generating a calibration plot and calculating the Brier score, calibration intercept, and calibration slope.

To determine the optimal cutoff value for calculating sensitivity and specificity, the Youden index along with its corresponding optimal sensitivity and specificity was computed. Additionally, a random forest model was developed as a sensitivity benchmark and compared with the logistic regression model. Decision curve analysis was subsequently performed to evaluate the model's range of threshold probabilities [22]. Finally, a nomogram was constructed based on the final model and the optimal clinical cutoff value, and its predictive performance was validated.

All data processing was done using R 4.4.3 software.

## 3 Results

### 3.1 Patient Characteristics

**FIGURE 1:**
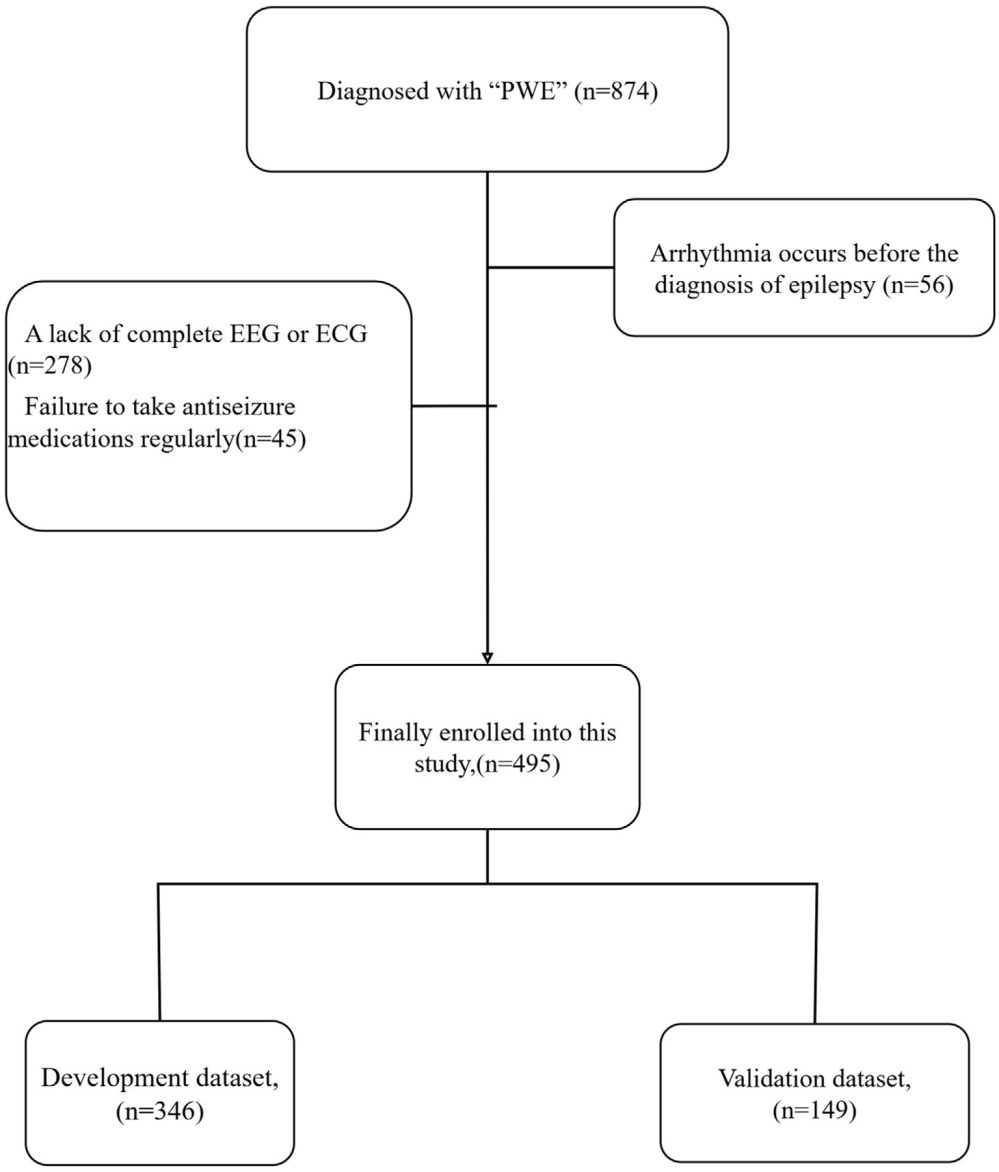
A total of 874 patients with epilepsy were initially identified. Following screening based on inclusion and exclusion criteria, 495 eligible participants were ultimately enrolled in the study. These subjects were subsequently randomly allocated to either the development dataset or validation dataset at a ratio of 7:3.

The clinical data of all PWE at the Affiliated Hospital of Qingdao University between January 2022 and February 2025 were collected, and 874 patients who met the inclusion criteria mentioned above were screened. A total of 379 patients were excluded because of the exclusion criteria mentioned above; data from 495 patients with PWE were finally included in this study for analysis. Using the random seed function in R, the data were randomly divided into a development dataset and a validation dataset in a 7:3 ratio. A total of 346 of the 495 patients were included in the development dataset; the other 149 patients were included in the validation dataset (Figure 1). Table 1 describes the characteristics of the development dataset, validation dataset, and the overall dataset.

**TABLE 1:** All variables for included patients are presented as follows: Quantitative data are expressed as mean ± standard deviation (SD), while categorical variables are reported as numbers (n) and percentages (%). Preliminary discrepancy analysis is presented in the final column of the table.

		Total (*n* = 495)	Without arrhythmia (*n* = 373)	Arrhythmias (*n* = 122)	
Gender	Female	213	178 (47.7%)	35 (28.7%)	2.27 (1.46–3.53, *p* < 0.001)
	Male	282	195 (52.3%)	87 (71.3%)	
Age	Mean ± SD	37.37 ± 18.17	36.2 ± 17.9	40.9 ± 18.7	1.01 (1.00–1.03, *p* = 0.014)
TBI or craniocerebral operations	No	430	331 (88.7%)	99 (81.1%)	1.83 (1.05–3.19, *p* = 0.033)
	Yes	65	42 (11.3%)	23 (18.9%)	
Smoke	No	470	358 (96%)	112 (91.8%)	2.13 (0.93–4.88, *p* = 0.073)
	Yes	25	15 (4%)	10 (8.2%)	
Hypertension	No	441	334 (89.5%)	107 (87.7%)	
	Yes	54	39 (10.5%)	15 (12.3%)	
Febrile seizure	No	464	350 (93.8%)	114 (93.4%)	
	Yes	31	23 (6.2%)	8 (6.6%)	
Course of PWE	Mean ± SD	6.87 ± 9.0	6.8 ± 9.0	7.2 ± 8.9	
Oxcarbazepine	No	371	294 (78.8%)	77 (63.1%)	2.17 (1.40–3.39, *p* < 0.001)
	Yes	134	79 (21.2%)	45 (36.9%)	
Carbamazepine	No	458	346 (92.8%)	112 (91.8%)	
	Yes	37	27 (7.2%)	10 (8.2%)	
Sodium Valproate	No	359	277 (74.3%)	82 (67.2%)	1.41 (0.90–2.19, *p* = 0.131)
	Yes	136	96 (25.7%)	40 (32.8%)	
Lamotrigine (LTG)	No	446	341 (91.4%)	105 (86.1%)	1.73 (0.92–3.23, *p* = 0.088)
	Yes	49	32 (8.6%)	17 (13.9%)	
Lacosamide	No	476	362 (97.1%)	114 (93.4%)	2.31 (0.91–5.88, *p* = 0.079)
	Yes	19	11 (2.9%)	8 (6.6%)	
Perampanel	No	438	332 (89%)	106 (86.9%)	
	Yes	57	41 (11%)	16 (13.1%)	
Levetiracetam	No	329	246 (66%)	83 (68%)	
	Yes	166	127 (34%)	39 (32%)	
Number of ASM	None	68	58 (15.5%)	10 (8.2%)	1.51 (0.72–3.15, *p* = 0.275)
	Monotherapy	257	204 (54.7%)	53 (43.4%)	
	Polytherapy	170	111 (29.8%)	59 (48.4%)	3.08 (1.47–6.47, *p* = 0.003)
PR interval (ms)	Mean ± SD	156.3 ± 23.6	155.6 ± 19.5	158.5 ± 33.2	
QRS duration (ms)	Mean ± SD	94.1 ± 12.1	92.7 ± 11.4	98.6 ± 13.2	1.04 (1.02–1.06, *p* < 0.001)
QT interval (ms)	Mean ± SD	382.4 ± 33.3	378.8 ± 28.1	393.5 ± 44	1.01 (1.01–1.02, *p* < 0.001)
Corrected QT interval (ms)	Mean ± SD	414.4 ± 24.9	414.4 ± 23.7	413 ± 28.2	
Frontal QRS‐T Angle (°)	Mean ± SD	30.8 ± 28.5	27.6 ± 22.3	40.6 ± 40.9	1.01 (1.01–1.02, *p* < 0.001)
EEG	Normal EEG	99	76 (20.4%)	23 (18.9%)	
	Abnormal background without epileptiform discharges	115	85 (22.8%)	30 (24.6%)	
	Epileptiform discharges	281	212 (56.8%)	69 (56.6%)	

Among all patients who met the inclusion criteria, the main (SD) age was 37.37 (18.17). Of these, 283 patients were male, accounting for 57.9% of the total. The course of PTE was 6.87 (8.98) years at the last follow‐up. Among all the patients included in the study, every valid ECG recording could be classified into one of the first four categories according to the protocol‐defined criteria. There were no cases of epilepsy that required additional discussion for qualification. Among all included patients, the incidence of arrhythmia was approximately 24.6% (122/495). We excluded 18 cases of sinus arrhythmia, considering them as negative outcomes because its presence is an indicator of good cardiovascular health [23]. In the data we have gathered, the types of arrhythmias we have documented encompass tachycardia, bradycardia, conduction blocks, and atrial arrhythmias (atrial premature beats and atrial fibrillation).

A preliminary univariate analysis was performed on the data. From Table 1, it can be observed that the data distribution in the development dataset and validation dataset is relatively uniform.

### 3.2 Risk Factor Analysis

**FIGURE 2:**
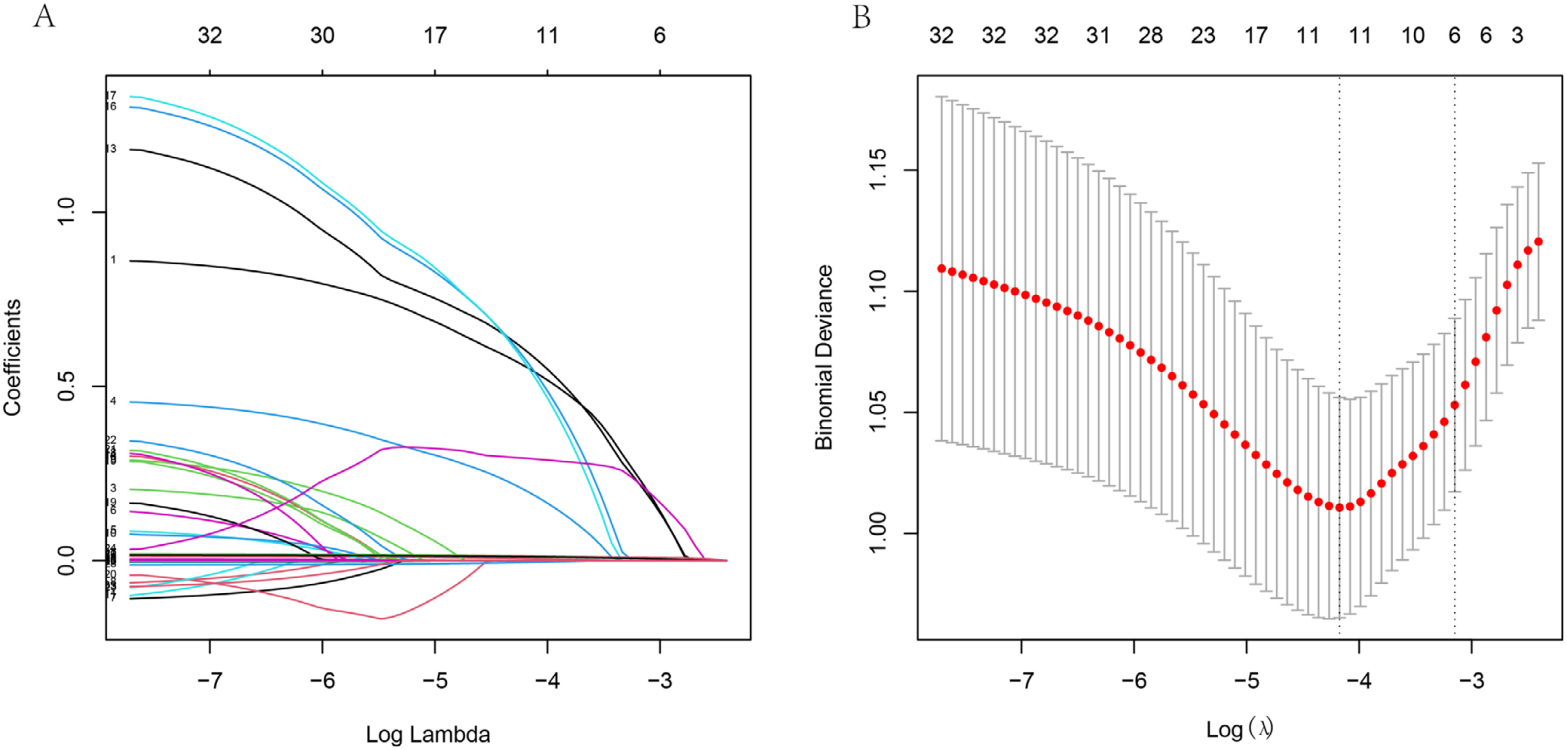
(A) Coefficient profile plot of LASSO regression. (B) Cross‐validation curve for LASSO regression analysis: At λ.min = 0.0154, 11 variables were selected; at λ.1SE = 0.0428 6 variables were selected.

We incorporated all the original variables into the LASSO regression analysis and performed 9‐fold cross‐validation (Figure 2). This process yielded the minimum value of lambda and the value at one standard deviation. Based on our sample size and event count, the final model was chosen at λ.1SE (λ = 0.0428), which included six variables, rather than at λ.min (λ = 0.0154), which included 11 variables. This decision was made to avoid an events‐per‐variable (EPV) ratio of less than 10, thereby enhancing the reliability of the coefficient estimates. The filtered variables are Gender, Oxcarbazepine, Number of ASM, QRS duration, QT interval, and Frontal QRS‐T angle, which were subsequently used to construct the logistic regression model.

### 3.3 Models Development and Validation

**FIGURE 3:**
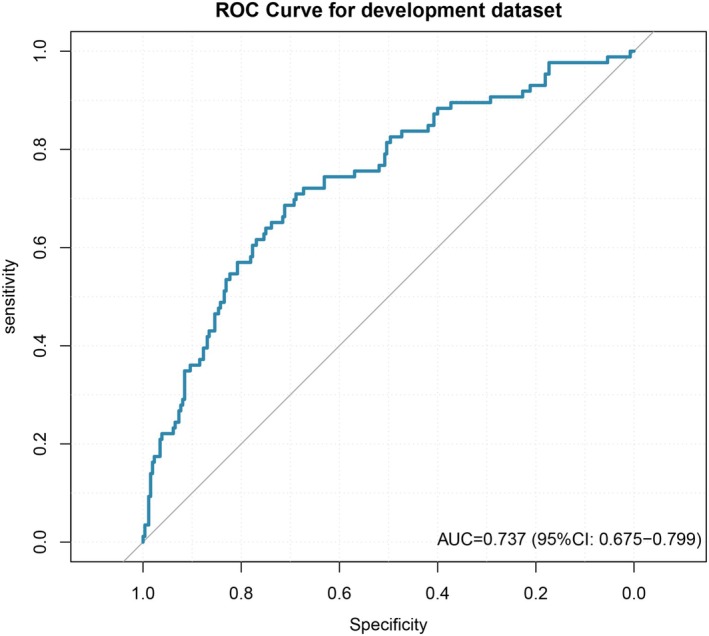
The ROC curve of the development dataset, with an AUC of 0.737 (95% CI: 0.675–0.799).

**FIGURE 4:**
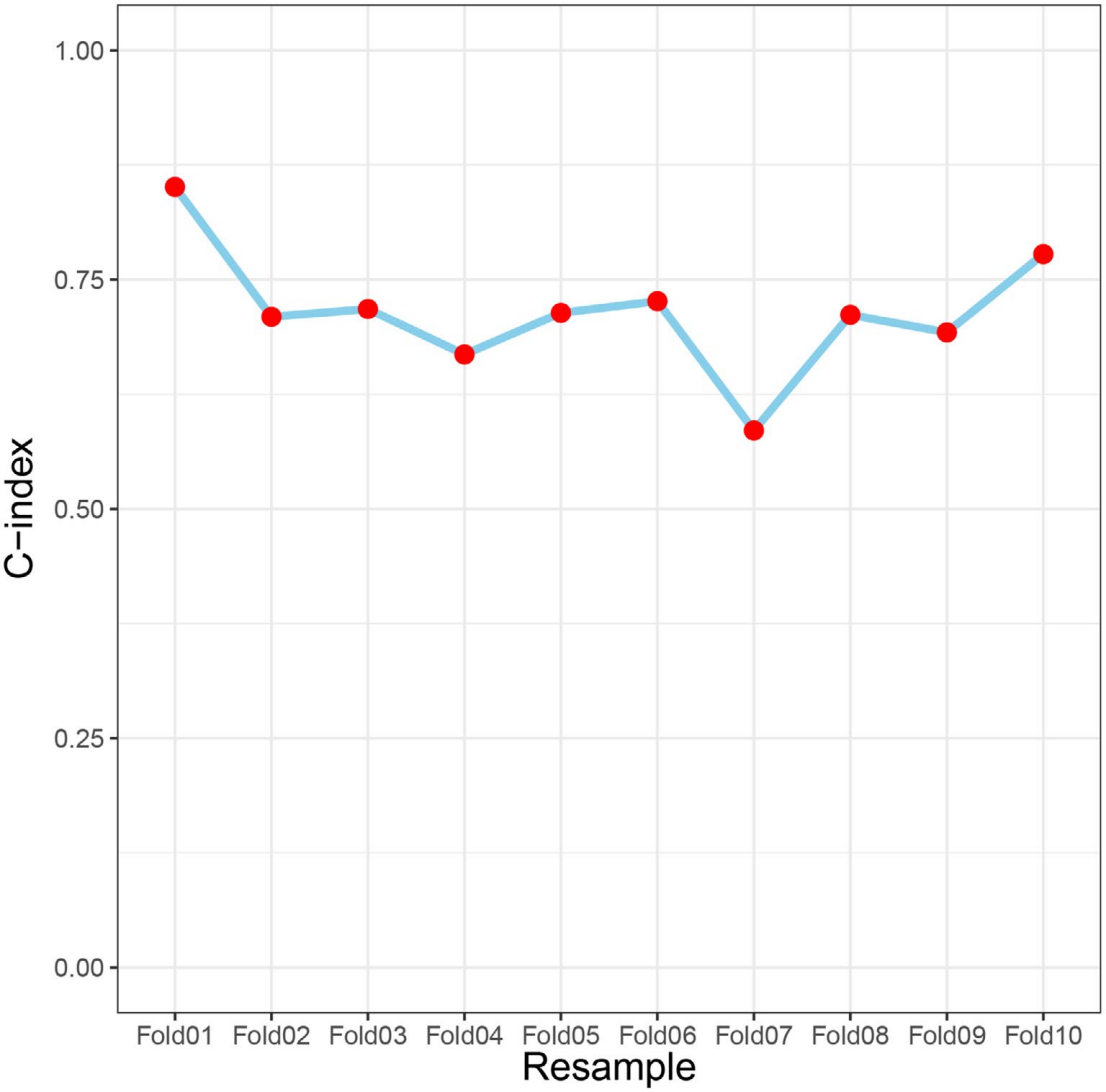
The visualization plot of the 10‐fold cross‐validation, with each point representing the C‐index corresponding to a resampling iteration.

**FIGURE 5:**
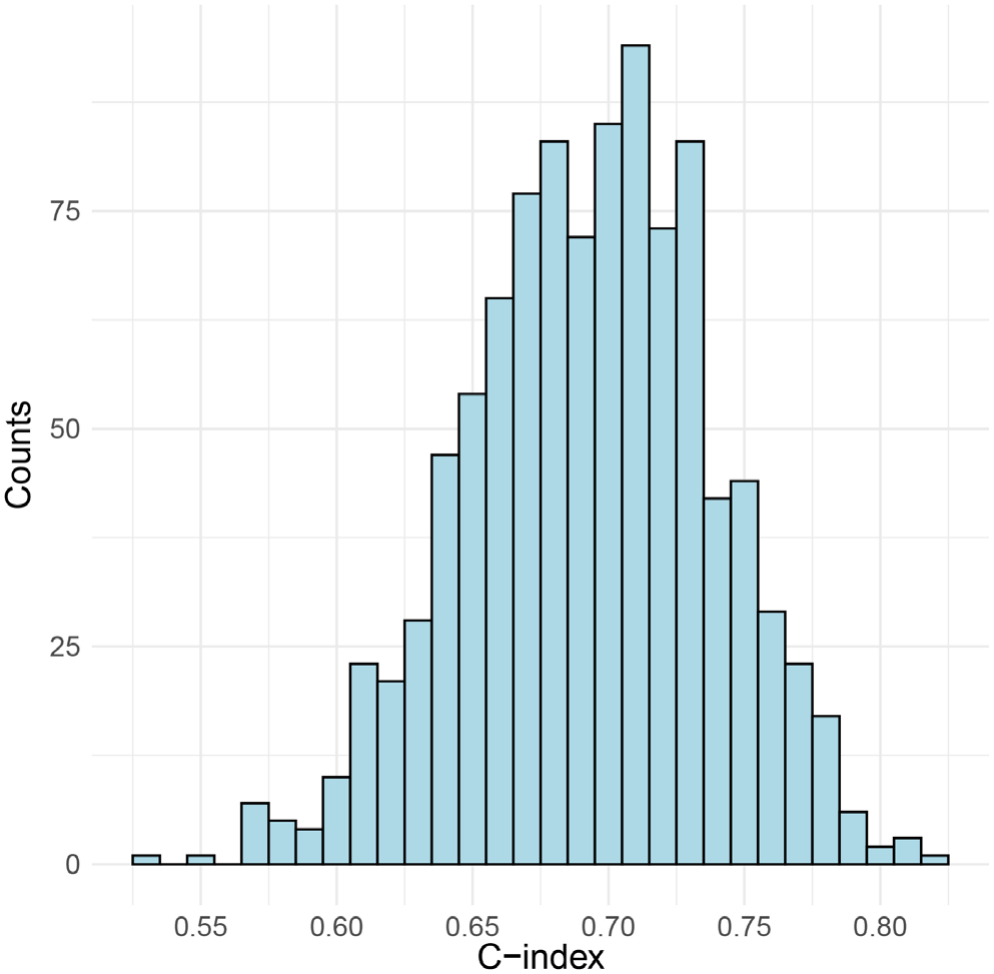
The visualization plot for the bootstrap optimism‐corrected validation with 1000 samples shows a C‐index of 0.694 (95% CI: 0.600–0.788).

The variables selected by the LASSO penalized regression were used to construct a logistic regression model. The initial calculated AUC of the model was 0.737 (95% CI: 0.675–0.799) (Figure 3). Subsequently, the Hosmer‐Lemeshow (H‐L) test was performed, yielding a *χ* ^2^ = 8.347, df = 8, and a *p* value = 0.400. For internal validation, k‐fold cross‐validation was applied to the established model; specifically, 10‐fold cross‐validation was conducted, and the corresponding plot was generated. The mean AUC from the 10 cross‐validation iterations was 0.711, which was close to the original model's AUC (Figure 4). Furthermore, 1000 bootstrap samples were used to estimate the shrinkage factor. After bootstrap optimism correction, the mean AUC and its 95% confidence interval were 0.694 (95% CI: 0.600–0.788) (Figure 5). The coefficients and intercept of this logistic regression model are presented in Table 2.

**TABLE 2:** The regression coefficients, intercept, and associated statistics for the logistic regression model are described.

	Estimate	Std.Error	z value	Pr (>|z|)
Gender	0.890098	0.317896	2.8	0.00511
Oxcarbazepine	0.343514	0.304008	1.13	0.2585
Number of ASMs	0.641935	0.232507	2.761	0.00576
QRS duration	0.011276	0.011876	0.949	0.3424
QT interval	0.017312	0.00431	4.016	0.0000591
Frontal QRS T angle	0.008461	0.004683	1.807	0.0708
Intercept	−10.6243			

	Estimate	Std.Error	z value	Pr (>|z|)
Gender	0.890098	0.317896	2.8	0.00511
Oxcarbazepine	0.343514	0.304008	1.13	0.2585
Number_of_ASM	0.641935	0.232507	2.761	0.00576
QRS_duration	0.011276	0.011876	0.949	0.3424
QT_interval	0.017312	0.00431	4.016	0.0000591
Frontal_QRS_T_angle	0.008461	0.004683	1.807	0.0708
Frontal_QRS_T_angle	0.008461	0.004683	1.807	0.0708
Intercept	−10.624278			

### 3.4 Models Validation

**FIGURE 6:**
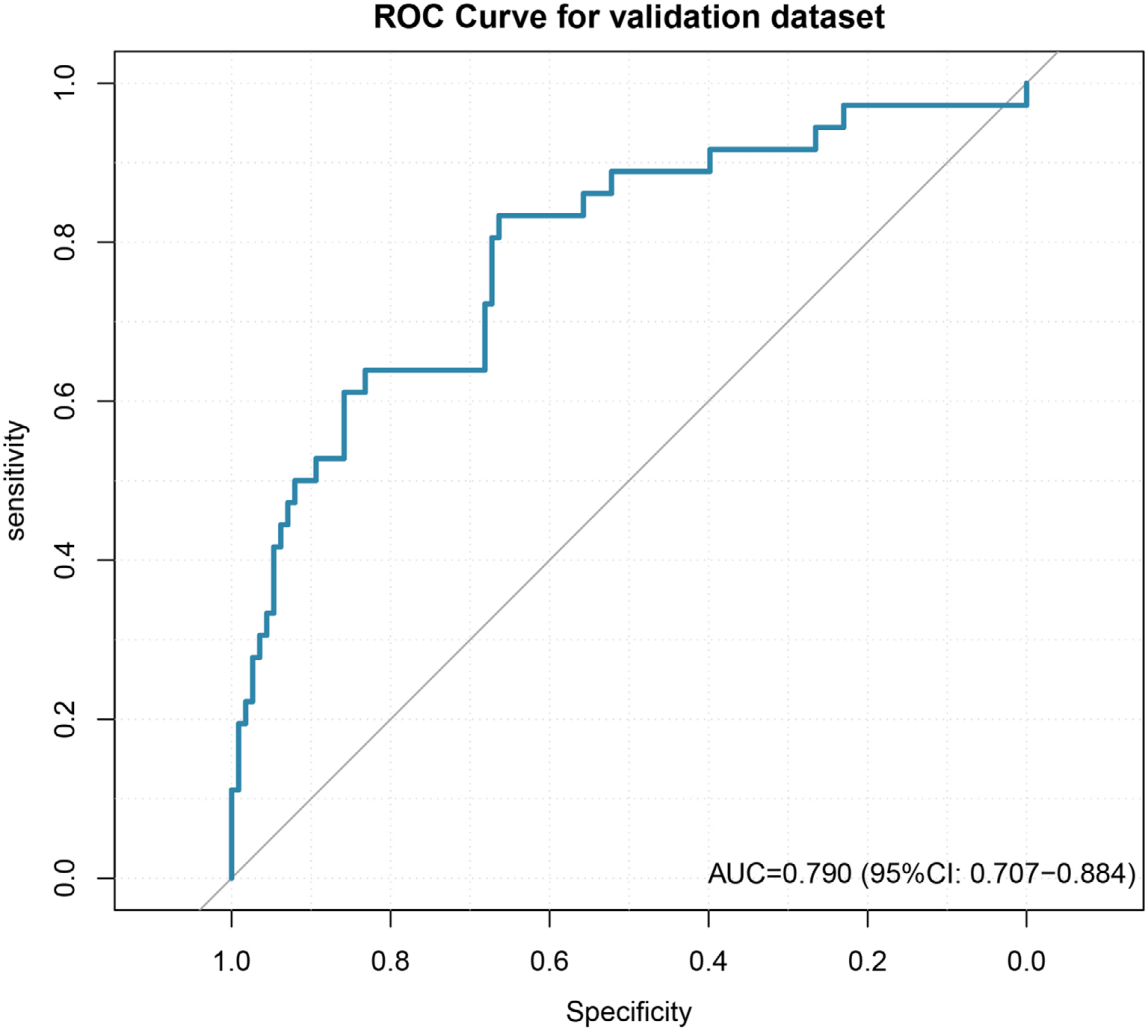
The ROC curve for the validation dataset, with an AUC of 0.790 (95% CI: 0.707–0.884).

The model was subsequently validated on the validation dataset, achieving an AUC of 0.790 (95% CI: 0.707–0.884) (Figure 6).

The logistic regression model is given by the following equation: y = −10.6243 + 0.890 × Gender+0.344 × Oxcarbazepine+0.642 × Number of ASM + 0.011 × QRS duration+0.017 × QT interval + 0.008 × Frontal QRS‐T angle.

**FIGURE 7:**
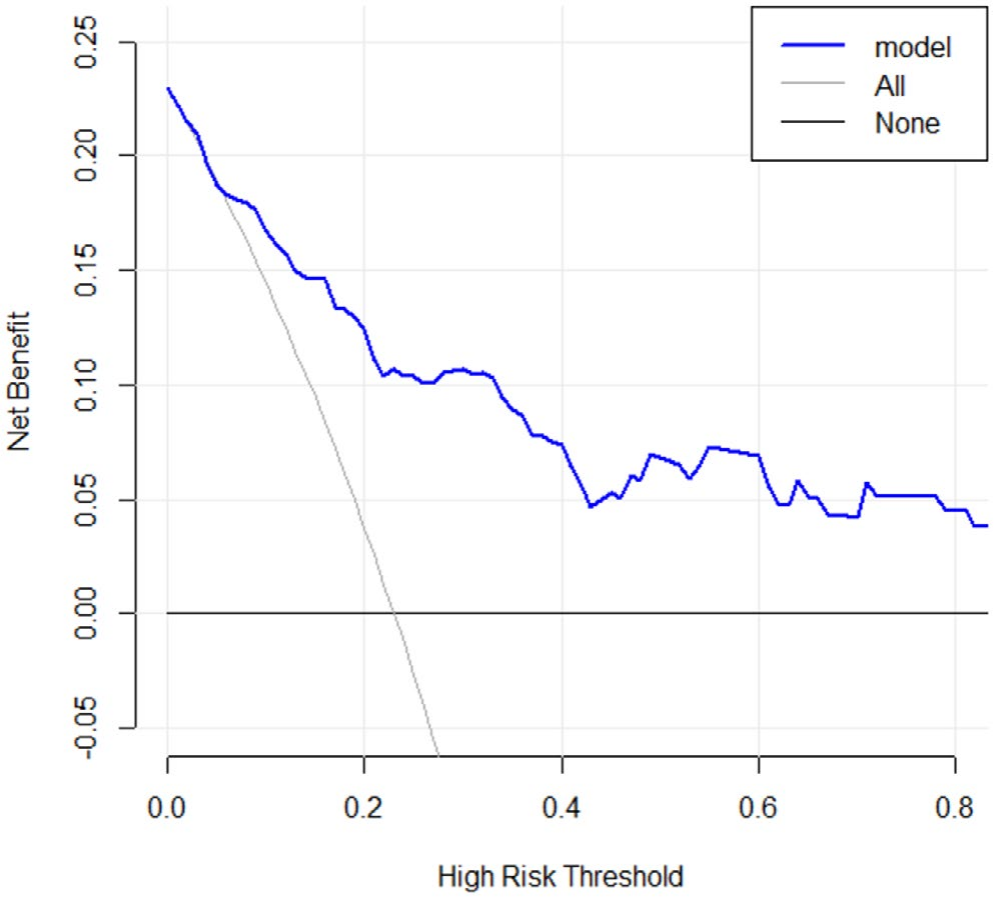
The clinical decision curve showed a significantly higher net benefit for the model compared to the baseline strategies when the threshold probability was between 0.1 and 0.8.

**FIGURE 8:**
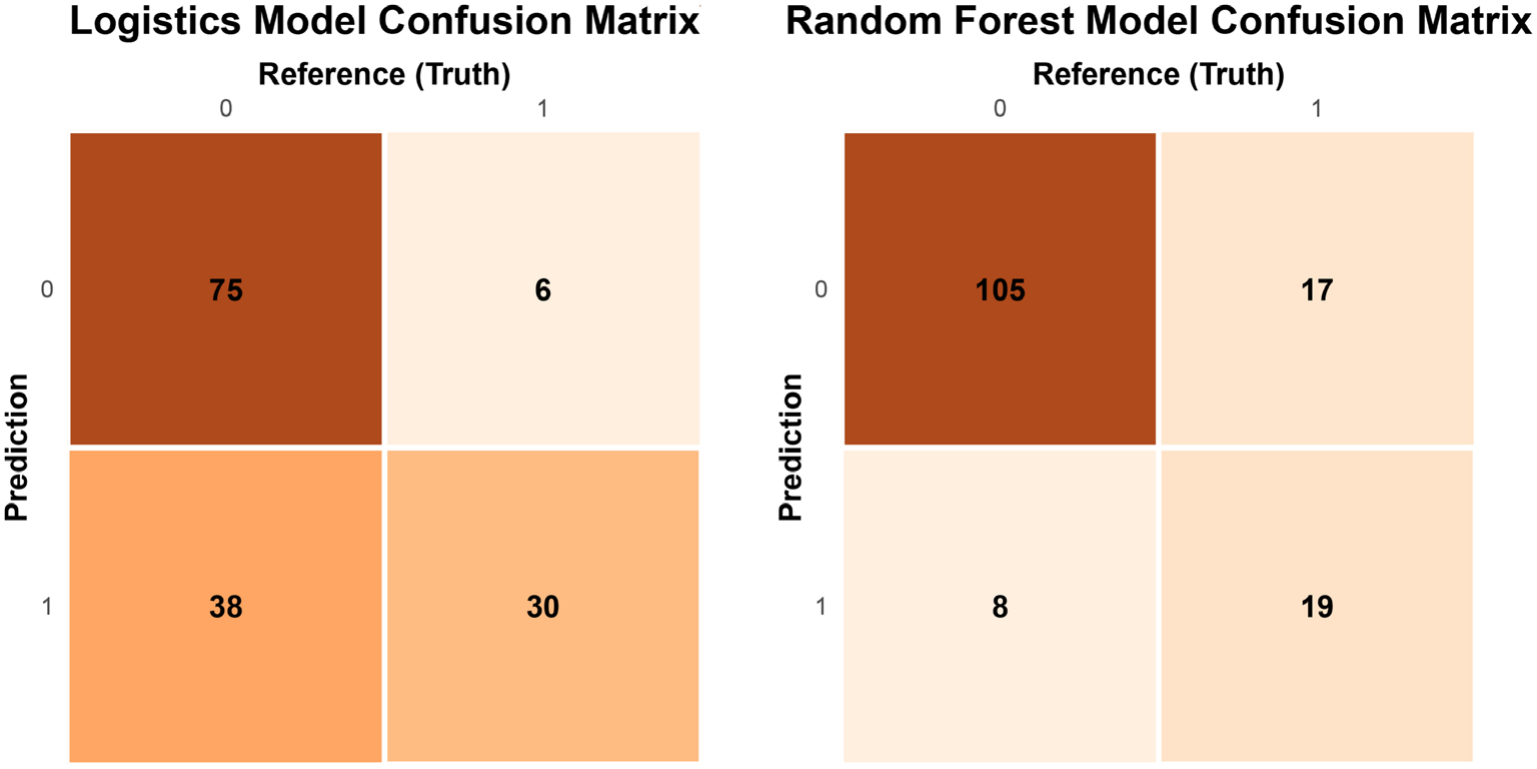
A comparative analysis of the confusion matrices for the validation set revealed that the logistic regression model achieved significantly higher sensitivity than the random forest model.

Furthermore, a Precision‐Recall (PR) curve was plotted, yielding a PR‐AUC of 0.63. To determine the optimal cut‐off value for calculating sensitivity and specificity, the threshold corresponding to the maximum Youden index was computed (threshold = 0.2427, specificity = 0.833, sensitivity = 0.664). The decision curve analysis for the validation dataset demonstrated that the model yielded a substantially higher net benefit than the baseline strategies when the threshold probability was between 0.1 and 0.8 (Figure 7). For sensitivity analysis, a random forest model was constructed as a benchmark. The threshold based on its Youden index corresponded to a specificity of 0.929 and a sensitivity of 0.528 (Figure 8), which indicates that the random forest model was compromised by both overfitting and suboptimal sensitivity.

**FIGURE 9:**
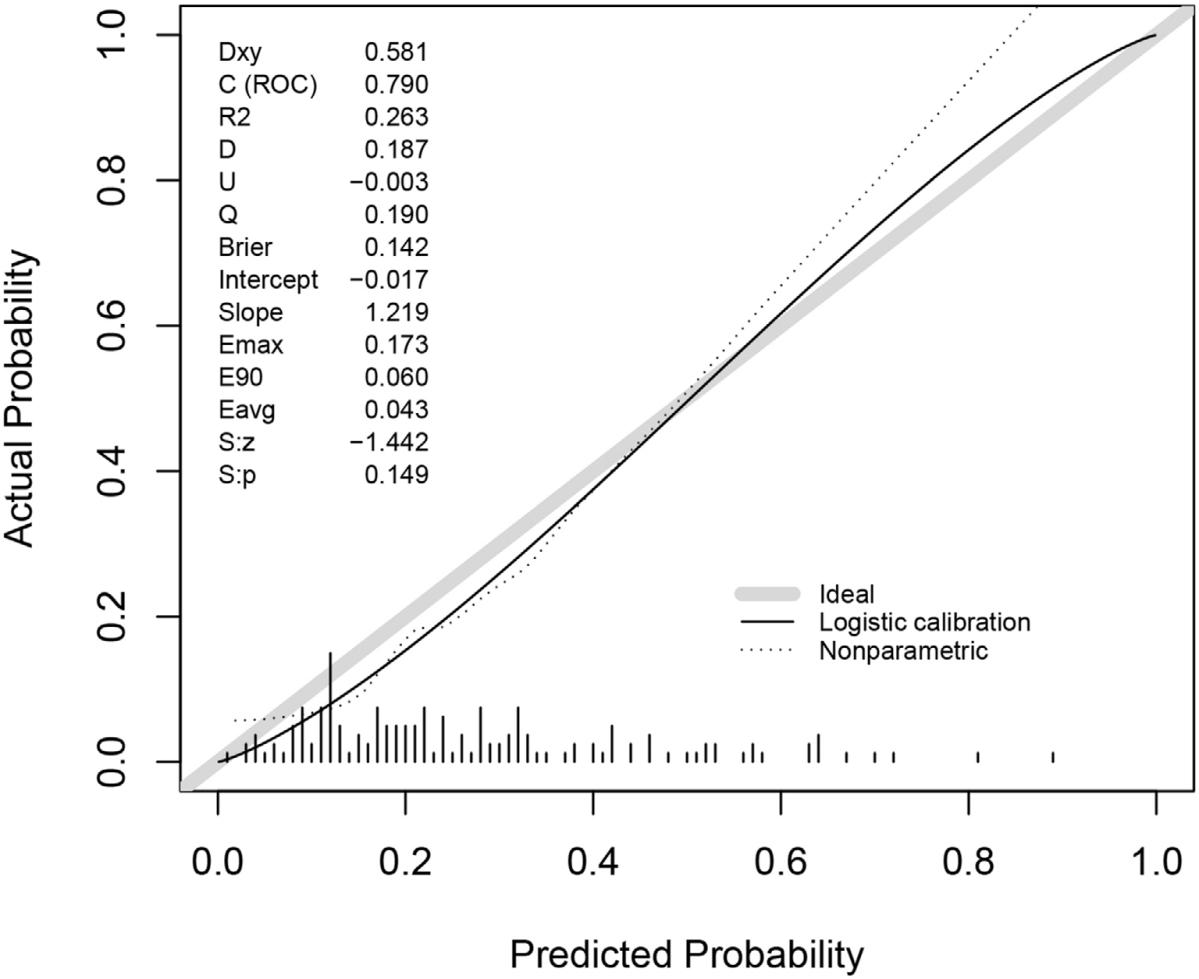
Calibration curves for the validation dataset of the Logistics model yielded a Brier score of 0.142, a calibration slope of 1.219, and an intercept of −0.017.

Furthermore, the model's calibration was assessed. The calibration curve evaluated the agreement between predicted and actual probabilities (Figure 9). Quantitatively, the model achieved a Brier score of 0.142, demonstrating good predictive accuracy. The calibration slope of 1.219 indicates that the model's probability estimates maintain a good dynamic range for risk stratification. While the calibration intercept of −0.017 points to a slight systematic overestimation of risk, the very small magnitude of this value confirms that the overestimation is not clinically significant.

### 3.5 Total Model and Nomogram

**FIGURE 10:**
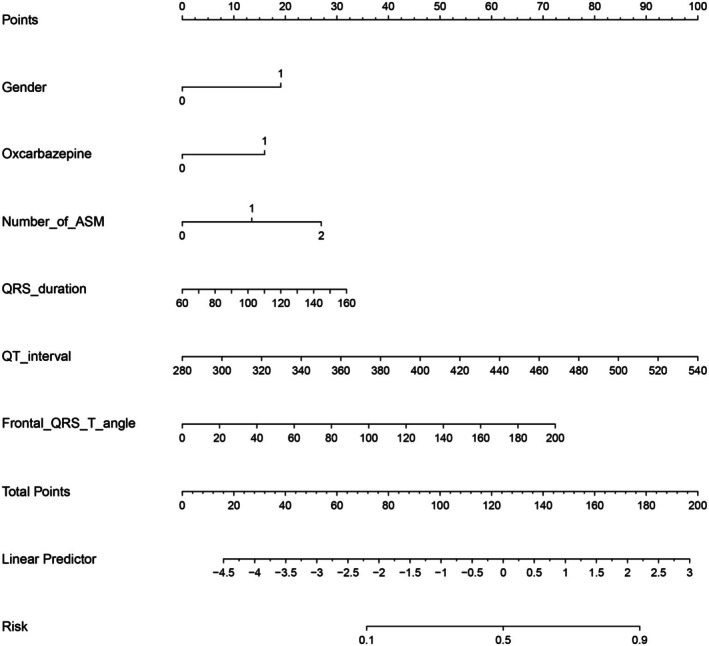
A nomogram derived from the Logistics Model was constructed to visually represent the predictive algorithm, wherein each variable was assigned a specific point score (total range: 0–200 points). The total score was subsequently converted to predicted probabilities ranging from 0.1 to 0.9 via a predefined risk stratification scale.

To present the final model performance on the full data, the logistic regression model was fitted to the entire dataset, resulting in an overall AUC of 0.752 (95% CI: 0.701–0.804). Subsequently, a nomogram was plotted to visualize the model for practical application (Figure 10). In our nomogram, each variable included in the analysis is assigned a corresponding score based on its weight in the model. This nomogram provides a convenient tool for predicting the probability of arrhythmia occurrence in epilepsy patients. For an individual epilepsy patient, we first determine the position of each variable on its corresponding axis. Then, we sum the scores of each variable to form a total score by drawing a line on the points axis. The total score axis, ranging from 0 to 200 points, is used to estimate the probability of arrhythmia occurrence for each given patient, with probabilities ranging from 0.1 to 0.9. The optimal cutoff threshold was determined to be 124 points, with scores equal to or exceeding this value considered positive results.

## 4 Discussion

Epilepsy is a chronic condition that can persist throughout a patient's lifetime, necessitating equal attention to both its complications and comorbidities [8]. In particular, there is growing concern regarding epilepsy combined with arrhythmia. Recent reviews have highlighted several associated mechanisms, including ictal bradycardia and asystole in temporal lobe epilepsy, ion channel disorders involving the heart–brain axis, ASMs‐induced arrhythmias, and central cardio‐respiratory autonomic dysfunction resulting from epileptic seizures, which may ultimately lead to SUDEP [8].

Among these factors, changes in the QT interval in PWE have been shown to possess predictive value for arrhythmias. In our univariate analysis and LASSO regression, the QT interval ranked first. This aligns with previous research findings that PWE with long QT syndrome are prone to malignant arrhythmias and adverse cardiac events [24]. Although the etiologies differ, they share the common feature of QT interval prolongation.

The investigation into the primary etiology linking epilepsy and arrhythmias often begins with genetic analysis. These conditions share multiple genetic loci associated with comorbidity, including various ion channel genes, mitochondrial genes, and others [13]. A recent systematic review on the genetics of SUDEP emphasized that variants in genes encoding sodium and potassium channel subunits are the most frequently implicated [25]. However, the detection rate of such variants in patients remains relatively low, at approximately 11%, and genetic testing entails high costs. These limitations constitute one of the principal reasons why genetic‐level analysis was not pursued in the present study.

ASMs can induce cardiotoxic effects through various pathways [8], with enzyme‐inducing ASMs and sodium channel‐blocking ASMs being particularly notable [26]. A Canadian longitudinal study revealed that active epilepsy patients treated with sodium channel‐blocking ASMs (NABs), including oxcarbazepine and lamotrigine, are more susceptible to developing cardiac conduction disorders (CCD) and arrhythmias [27]. It is noteworthy that sodium channel‐blocking ASMs are associated with a higher risk of arrhythmias, a point also highlighted in a study investigating the long‐term risk of epilepsy and arrhythmias [2]. This may provide supporting evidence for the concept of cardio‐cerebral channelopathies. In studies concerning lamotrigine, the cardiac safety profile of lamotrigine remains inconclusive [28]. An FDA analysis report indicates no significant difference in the incidence of arrhythmias and cardiac arrest events between lamotrigine and non‐sodium channel blockers [29]. A rapid systematic review also suggests that there is insufficient evidence to either support or refute an association between lamotrigine and sudden death or electrocardiogram changes in patients with or without epilepsy [30]. A real‐world observational study indicated a strong correlation between lamotrigine and the occurrence of ventricular tachycardia [31]. Additionally, an animal study further demonstrated a high incidence of lamotrigine‐induced ventricular tachycardia [32]. In the literature on dose‐dependent effects of lamotrigine, case reports have documented that supratherapeutic doses of lamotrigine can lead to severe cardiotoxicity [33]. However, therapeutic doses of lamotrigine did not prolong the QT interval in healthy subjects [34]. Lacosamide exerts its antiepileptic effect by selectively enhancing sodium channel slow inactivation [35]. However it also inhibits the cardiac sodium channel SCN5A, which may represent a potential mechanism for arrhythmias [36]. The risk of arrhythmias associated with lacosamide has been previously highlighted in our meta‐analysis. In a study investigating the safety profile of lacosamide, instances of bradycardia and QT interval prolongation attributed to lacosamide were reported [37]. Regarding QTc, a retrospective cohort study demonstrates that a prolonged optimal cutoff QTc interval is predictive of all‐cause mortality in patients evaluated for seizure and those diagnosed with epilepsy. The study advocates for the use of 12‐lead electrocardiograms in the assessment of epilepsy patients [38].

In our retrospective study, it was found that gender had a significant effect during both variable screening and model construction. Within our research cohort, 35 females (16%) were diagnosed with arrhythmias, compared to 87 males (30%), suggesting that male epilepsy patients may have a greater susceptibility. A fundamental experimental study on gender and arrhythmias has revealed that gender significantly influences the occurrence of arrhythmias during the aging process, particularly in males. It also points out that the incidence of arrhythmias in males is associated with excessive catecholamine stimulation, whereas females exhibit a greater susceptibility to arrhythmias in conditions of increased oxidative stress [39]. Another related study indicates a marked elevation in serum and cerebrospinal fluid catecholamine metabolites flowing from complex partial seizures and generalized tonic–clonic seizures [40]. In the concept of epileptic heart proposed by Richard L. Verrier, long‐term chronic seizures lead to repeated stimulation by catecholamines and hypoxemia, ultimately resulting in impaired cardiac function [41, 42, 43]. Another study on arrhythmias in female patients mentions that women are more prone to atrioventricular nodal reentrant tachycardia, while men have a predisposition to atrioventricular reentrant tachycardia. This gender disparity is associated with the cardiac conduction structure, the influence of circulating hormones, and intrinsic electrophysiological sex differences [44]. Meanwhile, we have found a positive correlation between age and the incidence of arrhythmias. Research indicates that the aging process can lead to the development of a pro‐arrhythmic substrate within the myocardium, which is more commonly observed in males [39]. Another study provides a molecular‐level analysis of the correlation between age and arrhythmias, noting that collagen content in the heart increases with age; age‐related inflammation can lead to myocardial remodeling, among other changes [45].

Furthermore, PWE who also suffer from traumatic brain injury, a condition known as post‐traumatic epilepsy, are at a higher risk of developing arrhythmias [46]. The extracranial complications of traumatic brain injury have been reported for some time, with QTc prolongation and supraventricular arrhythmias being the most common among cardiac complications [47]. The exact cause of arrhythmias in these patients cannot be determined with certainty; it may be due to a combination of factors related to both epilepsy and traumatic brain injury. However, in our study, the risk of arrhythmias in this group of patients is indeed higher. Regarding the duration of epilepsy, our data do not support the notion that a longer course of the disease equates to a higher risk, although it is indeed a risk factor for SUDEP [48]. The frontal QRS angle emerged as a distinctive predictor in our analysis. It demonstrated a significant association in univariate screening (*p* < 0.001), was retained through the LASSO penalty regression for model construction, and accounted for a considerable proportion of the total points in the final nomogram. The utility of the frontal QRST angle, an indicator of cardiac repolarization, in predicting adverse cardiac events has been documented in a study on epilepsy prediction [49]. Additionally, the QRST angle, which quantifies the heterogeneity of depolarization and repolarization, significantly contributes to the diagnosis and prognosis of patients with suspected non‐ST‐segment elevation myocardial infarction [50]. This is corroborated by another two studies reporting an association between the spatial QRS‐T angle and cardiac autonomic neuropathy in patients with type 2 diabetes [51, 52]. Integrating these findings with the significant intergroup differences in the frontal QRS‐T angle observed in our study, it is plausible that epilepsy may influence cardiac autonomic rhythm and myocardial repolarization through autonomic nervous pathways. The QRST angle may serve as a sentinel indicator of this interaction. The predictive capability of this variable for arrhythmias in epilepsy patients warrants further investigation.

There is a growing trend in the use of long‐term electrocardiogram (ECG) and electroencephalogram (EEG) monitoring for epilepsy patients, exemplified by devices such as subcutaneous implants for monitoring heart and seizure activity, biometric smart shirts, and highly integrated ear‐worn devices that detect both brain and heart electrical activity [53, 54, 55].

Limitations of the current study: Limited sample size: Although we initially collected 800 cases, a significant proportion of patients lacked complete electrocardiographic (ECG) monitoring data, resulting in analyzable sample attrition and potential selection bias. Single‐center design: Only internal validation was performed, and the lack of external validation requires further verification of the model's generalization ability. Future studies should conduct external validation in multi‐center or prospective cohorts. Retrospective nature: Our data collection did not include important socioeconomic variables (e.g., financial status, educational level, family background) or incorporate brain MRI findings in the analysis. ECG data limitation: The analysis was restricted to interictal ECG recordings, with no available ictal (seizure‐onset) ECG data. Furthermore, the sources of ECG data exhibited a certain degree of heterogeneity, which may introduce potential bias. Future prospective studies may be necessary to properly address and minimize this potential bias.

Proposed solutions and future research directions include the following: First, large‐scale, multicenter prospective cohort studies should be implemented to enhance sample representativeness and data completeness. Second, novel wearable ECG monitoring technologies should be utilized to concurrently capture both ictal and interictal electrophysiological data.

## 5 Conclusions

The occurrence of arrhythmias in epilepsy patients involves multiple factors, including gender, use of ASMs, QT interval changes, and traumatic brain injury, among others. Our study developed a predictive model that demonstrates good performance in assessing the risk of arrhythmia comorbidity in epilepsy patients, providing a novel approach for monitoring this condition. The clinically applicable nomogram developed based on this model can assist clinicians in the long‐term management of patients with epilepsy.

## Author Contributions

Y.L. conceived the idea, collected the data, and was the major contributor in drafting and writing the manuscript. Z.S. and S.S. analyzed the data and contributed to drafting the manuscript. J.Z. and Y.S. supervised the research and revised the manuscript. All authors read and approved the final version.

## Funding

This work was supported by the National Natural Science Foundation of the People's Republic of China (Grant no. 82071453) and Natural Science Foundation of Shandong Provincial (Grant no. ZR2025MS1280). At the same time, this work is also supported by the Qingdao Healthcare Elite Talent Development Program.

## Disclosure

The authors have nothing to report.

## Consent

Our study was conducted in accordance with the ethical standards of the Declaration of Helsinki and its later amendments. The study was approved by the Ethics Review Committee of the Affiliated Hospital of Qingdao University, with the ethical approval number QYFY WZLL 30213.

## Conflicts of Interest

The authors declare no conflicts of interest.

## Data Availability

The data that support the findings of this study are available from the corresponding author upon reasonable request.
